# Secure Data Aggregation Based on End-to-End Homomorphic Encryption in IoT-Based Wireless Sensor Networks

**DOI:** 10.3390/s23136181

**Published:** 2023-07-06

**Authors:** Mukesh Kumar, Monika Sethi, Shalli Rani, Dipak Kumar Sah, Salman A. AlQahtani, Mabrook S. Al-Rakhami

**Affiliations:** 1 Panipat Institute of Engineering and Technology, Panipat 132103, Haryana, India; mukeshk.chawla@gmail.com; 2 Chitkara University Institute of Engineering and Technology, Chitkara University, Rajpura 140401, Punjab, India; 3 Department of Computer Engineering and Applications, GLA University, Mathura 281406, Uttar Pradesh, India; 4 Department of Computer Engineering, College of Computer and Information Sciences, King Saud University, P.O. Box 51178, Riyadh 11543, Saudi Arabia; 5 Department of Information Systems, College of Computer and Information Sciences, King Saud University, P.O. Box 51178, Riyadh 11543, Saudi Arabia

**Keywords:** homomorphic encryption, data aggregation, wormhole attack, secure data aggregation, IoT-based WSN

## Abstract

By definition, the aggregating methodology ensures that transmitted data remain visible in clear text in the aggregated units or nodes. Data transmission without encryption is vulnerable to security issues such as data confidentiality, integrity, authentication and attacks by adversaries. On the other hand, encryption at each hop requires extra computation for decrypting, aggregating, and then re-encrypting the data, which results in increased complexity, not only in terms of computation but also due to the required sharing of keys. Sharing the same key across various nodes makes the security more vulnerable. An alternative solution to secure the aggregation process is to provide an end-to-end security protocol, wherein intermediary nodes combine the data without decoding the acquired data. As a consequence, the intermediary aggregating nodes do not have to maintain confidential key values, enabling end-to-end security across sensor devices and base stations. This research presents End-to-End Homomorphic Encryption (EEHE)-based safe and secure data gathering in IoT-based Wireless Sensor Networks (WSNs), whereby it protects end-to-end security and enables the use of aggregator functions such as COUNT, SUM and AVERAGE upon encrypted messages. Such an approach could also employ message authentication codes (MAC) to validate data integrity throughout data aggregation and transmission activities, allowing fraudulent content to also be identified as soon as feasible. Additionally, if data are communicated across a WSN, then there is a higher likelihood of a wormhole attack within the data aggregation process. The proposed solution also ensures the early detection of wormhole attacks during data aggregation.

## 1. Introduction

The IoT-based wireless Sensor network (WSN) is a revolutionary system for smart observation. An IoT-based Wireless Sensor Network (WSN) is defined as a number of spatially dispersed and dedicated sensors for observing and recording the physical conditions, such as temperature, humidity, etc., of the environment. The collected data are forwarded through a wireless network to an internet-based base station. The primary goal of data fusion or aggregation is to extend network life by reducing sensor network resource use, which includes batteries, power, and bandwidth [1,2]. Data aggregation techniques, on the other hand, could affect key quality of service measures in WSN, such as accuracy, speed, and failure [3]. Furthermore, data aggregation introduces new risks. A hacked sensor node, for instance, might either fraudulently release or broadcast the data it acquires from neighbouring nodes, or return random results as aggregated data. As a result, an opponent may violate both the secrecy and the integrity of the information over a broad section of the WSN by compromising a significant number of aggregating units near the base station. Rafik et al. [4] offered a secure data accumulation strategy which guarantees data privacy through symmetric-key homomorphic encryption (HE) using homomorphic signatures to validate data integrity. The protocol [4] is prone to wormhole attack. Lacking understanding about the key procedures of cryptography, a wormhole exploits the network communication architecture. Wormhole threats are primarily designed to confuse routing protocols and communication services [5]. As a result, developing an end-to-end secure data aggregation technique, while achieving confidentiality, integrity and attack detection, is a challenging task because an effective security strategy is essential in order to preserve integrity and durability and to retain sensitive data. The key objectives of the proposed protocol are as follows.

i. A novel HE technique enabling end-to-end data secrecy/confidentiality is proposed. The proposed EEHE could be used by aggregators to apply arithmetic aggregation functions on cipher texts.
ii. MAC is used to ensure data integrity. Within the proposed methodology, monitoring nodes generate MACs to the collected data so that certain participants in the group may instantly derive and check the MACs to ensure data integrity. As a result, there is no need to provide the non-encrypted data for confirmation.
iii. To identify wormhole attacks as soon as feasible during the data forwarding and aggregating operations, a paradigm focused upon neighbouring tables is proposed, comprising a monitoring, forwarding, and an aggregator’s adjacent node.

## 2. Related Work

The literature discusses a vast scope of secure data aggregation methodologies centered around homomorphically encrypted algorithms. Hung et al. suggested a solution [6] that guarantees the confidentiality of data, reliability, and resistance to eavesdropping threats. Additionally, it recognizes malicious activity with certain added costs. The scheme’s primary issue is that no source authentication is accomplished, making it susceptible to Sybil threats. The above limitation is taken into account in the concealed data aggregation (CDA) method [7] in which data aggregators (DA) perform only gathering and merging operations using encrypted messages. As a result, DAs are not required to hold vulnerable encryption keys. The concept of clustering along with data aggregation was introduced by the authors of the protocol [8], in which the notion of clustering, as well as data aggregation and algebraic features of polynomials, was included. A further end-to-end data aggregating strategy [9], which employs homomorphic encryption includes elliptic curve cryptography. Compared with previous complicated techniques, the elliptic curve cryptography technique allows nodes to produce keys with a reduced key size. This technique is remarkable for generating two distinct encrypted messages given two exactly identical messages. This is robust to documented plain text threats, and man-in-the-middle attacks. However, such a solution merely gives secrecy, neither authentication nor integrity. In order to achieve integrity and authentication along with confidentiality, there was a need for more efficient secure data aggregation techniques. Authors [10] suggested a simple and evidently secure encrypting approach relying upon indistinguishable characteristics of a cryptographic basic, Pseudo Random Function (PRF). The methodology assumes the integrity of aggregated data, but also end-to-end authentication. The fundamental aspect of the next protocol, SEEDA, is that it has minimal network communication overheads. The constraint of such an approach is that it only achieves secrecy and does not ensure integrity or authentication. Another issue, along with the achievement of confidentiality, integrity and authentication, is that the data aggregation process is vulnerable to various attacks, such as false data injection, node compromised attacks, Sybil and wormhole attacks. Data aggregation incorporating fake information monitoring is enabled by a protocol [11]. To achieve this, the monitored nodes from every Data Aggregator (DA) not only perform data aggregation but also calculate the appropriate MAC for data verification at respective allocated paired members during the design stage. To ensure data exchange confidentiality, the sensor nodes in between subsequent DAs verify the data integrity of the encrypted information instead of the raw text. The technique [12] was originally designed for multilayer data gathering and employs various keys for data encryption. For data integrity and secrecy, it utilizes elliptic curve cryptography and HE. This is resilient to replay vulnerabilities, eavesdropping, recognized simple text attacks, encrypted analysis, and illegal aggregation. Energy Efficient Hierarchical Aggregation (EEHA) achieved confidentiality and integrity, but it only addressed eavesdropping and replay attacks [13]. SEDA-ECC [14] achieves only confidentiality with the help of ECC (Elliptic Curve Cryptography), but the main focus was on the compromised node attack. The protocols [15,16,17,18,19,20] achieved confidentiality, authentication and integrity, along with addressing of false data injection attacks, snooping and man in middle attacks with the full HE scheme. Researchers in [21,22,23,24,25,26] have proposed various quality of service (QoS) metrics, but without securing the data transmission that can be blocked in IoT-based WSN networks. In response to waves in the ocean, aquatic creatures, or ships that are passing, sensors in underwater WSN may sway up to 3 m/s. The movement of nodes and networking disconnections have not been taken into account in the node installation and localization strategies previously in operation. To overcome this issue, a dynamic topology control algorithm for node deployment (DTCND) in mobile UWSN was suggested in [27]. For underwater intelligent traffic control and underwater vehicle navigation systems, it is also regarded as a crucial and difficult component. The researcher group in [28] offers an overview of the fundamental ideas behind multisensor data fusion and a thorough analysis of the most widely used data fusion architectural models for marine wireless sensor networks. In [29], different routing protocols—AODV, DSR, and WRP—are analyzed. Two possibilities that depend on the number and movement of nodes have been taken into consideration. PDR and throughput both improve as the number of nodes increases. Furthermore, less delay was caused by low node density. In both instances, AODV had a greater packet delivery ratio and throughput, while WRP had the least delay. The efficiency of the ad hoc network was determined by the authors’ analysis of the average energy usage and the optimum routing protocol that was determined by the outcome. The researchers used IOT with WSN to recognize and transfer the data [30]. With cloud-assisted healthcare WSNs, a secure and reliable certificateless publicly monitored approach that provides adaptive data exchange and confidentiality control, as well as effective grouped user revoking, was introduced in [31]. Table 1 presents a comparative review of the numerous methodologies suggested for secure data aggregation. The analysis is focused around the following parameters: data confidentiality, data integrity, provider authentication, node reliability, attack prevention, aggregating functionality, and strategies adopted to accomplish secure data aggregation. Thus, according to Table 1, there have been diverse secure data aggregation methods that resolve the challenges of authentication, confidentiality, integrity, and threats such as sensor node compromised attacks, misleading data injectors, replay attacks, spoofing, defined simple text threats, encrypted assessment, and unauthorized access collation. However, none of the existing protocols addressed the problem of wormhole attacks in data aggregation. As a result, a novel data aggregation architecture that enables end-to-end data protection, verification, durability, and includes wormhole attack monitoring, is needed. The novel strategy must be reliable and effective in respect to power utilization, propagation latency and aggregation output accuracy. The proposed protocol in this research is an improvement over the existing protocol [4]. The methodology of Othman et al. [4] is founded around symmetric key HE to ensure data privacy, as well as applying a homomorphic signature to validate the aggregated data integrity. This could efficiently ensure data privacy, validate data, and obtain greater transmission performance. The proposed protocol is based on an asymmetric key HE, and asymmetric key techniques are more efficient in terms of key distribution, security level, and security services as compared with symmetric key techniques [5]. In an asymmetric key technique, integrity, authentication and non-repudiation are achieved along with data confidentiality. The existing protocol [4] is vulnerable to wormhole attack, as wormhole attack does not depend on strong cryptographic keys. Wormhole attacks can be carried out by a solitary or perhaps a couple of cooperating nodes, as well as by two or even more intruders associated by a high channel link known as a wormhole link.

## 3. Background, Network Architecture and Objectives

The protocol proposed in this paper uses end-to-end encryption, namely, EEHE based on asymmetric primitives. In this article, we first offer an brief overview of each of these aspects, along with additional cryptographic techniques, prior to discussing our modeling techniques and architectural aims.

### 3.1. Network Architecture

**Figure 1:**
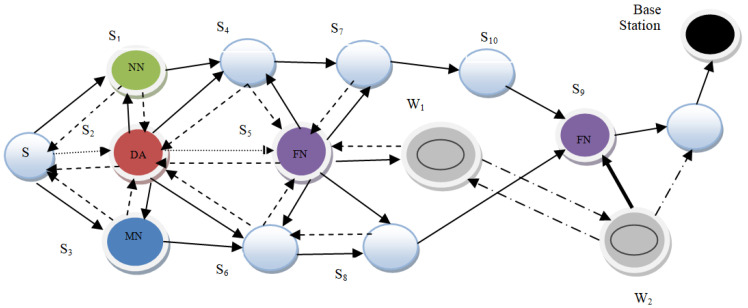
Proposed Architecture.

In the proposed architecture, a small part of the WSN, consisting of 15 sensor nodes, is considered. Out of these nodes, three nodes are designated as the Monitoring, Neighbouring, and Forwarding nodes (MNF), respectively. These nodes constitute a group known as the MNF group. The function of the monitoring node is to calculate the MAC of the data. The relaying and neighbouring nodes collaborate to verify the data generated by the monitoring node. The monitoring and neighbouring nodes record wormhole attacks as well. The DA gathers the information from every cluster, encodes it, and transmits it to a centralized controller, i.e., a base station. Every group’s forwarding node (FN) is also interconnected to the FNs of other groups. For fake information detection, each successive DA shares a symmetric key pair. Nodes W1 and W2 form a wormhole link in order to perform malicious activities such as packet dropping, data modification, routing misguiding, etc., as shown in Figure 1. The different colors of various nodes signify the nodes’ different functions; red is for the Data Aggregator node, purple is for the forwarding node, light blue is for the normal sensor node, dark blue is for the monitoring node, black is for the base station, and green is for the neighbouring node, respectively.

### 3.2. Terminology Used

In the proposed algorithm, terminology is given below:

DA = Data Aggregator, i.e., DA1, DA2……DAn

DA b = DA Backward

DA c = DA Current

DA n = DA Next

BS = Base Station (data transmission through the cloud or real-time assessment)

S = Sender node

MN = MNFs group’s monitoring nodes

NN = MNF group’s neighbouring nodes

FN = MNF group’s forwarding nodes

Gk = MNF group’s group key

EEHE(X) = End-to-End node x’s homomorphic value

Sub MAC = node x’s subMAC (Message Authentication Code) value

Sub MAC (EEHE(x)) = subMAC value x

KP = Public Key

KS = Secret Key

CNT = Common Neighbour Table

Mreq = request message

Mrep. = reply message

### 3.3. Attack Model

In the proposed algorithm, consideration is given to eavesdropping, false data injections and wormhole attacks. These attacks are mitigated with the help of HE, MAC and the neighbouring table of the MNF group, respectively.

### 3.4. MNF Group Formation

**Figure 2:**
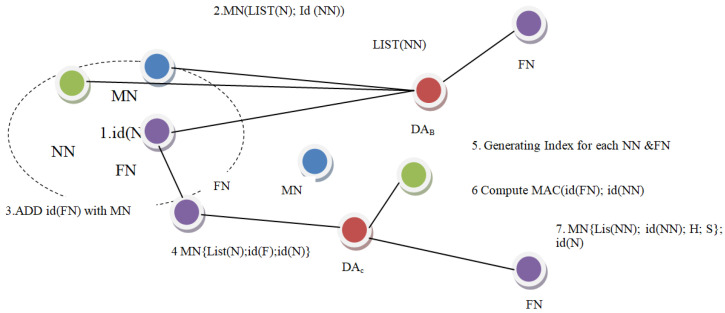
Process for MNF group formation.

The steps followed in the MNF group formation are shown in Figure 2. In the first step, the DAn computes the MAC ID (NN) and adds in the MAC list of (NN). In the second phase, a whole message M is transferred across two DAs via FN. The message M comprises the necessary information: list (NN) and MAC ID (NN). Subsequently, the Group-A FN relays such M packets to another associated FN, which is situated amid two aggregators. The FN inserts its own ID in packet M at the third stage. M contains M list (NN); MAC ID (NN); and ID (FN) in the fifth step. The IDs of all interconnected NN and FN between the DA, DAc and DAn become available to the next DA and DAn. In the next phase, the existing DA and DAc index all NN and FN node IDs. Index 1 to h includes the IDs of NNs, whereas index 1 to s includes the IDs of FNs. The monitoring node evaluates the MAC IDs of the NN and FN in the next phase. Once the data are transmitted to the Backward DA (DAB) in the seventh step, the DAB receives M with the value list M (NN); ID (NN), h, s. The procedure is to cover all the nodes. Each group’s monitoring node (MN) attached to the DA chooses this index and makes determinations about its own group members using combined FN and NN IDs [15].

#### E-E Homomorphic Encryption

Now, at the group level, encryption is performed utilizing a public key (Kp). The data are acquired in the format of cipher text as a result of this encryption. If an invader or adversary gains exposure to data, the data are meaningless or in an incomprehensible form. HE enables cipher text addition and multiplication. Assuming that m1 and m2 are two plain texts, and *, x are the homomorphic operations on the cipher texts and plain texts, respectively, we derive EEHE (m1)*EEH (m2) = EEHE (m1xm2), where EHEE (m) is the cipher text of m [18].

## 4. End-to-End Homomorphic Encryption-Based Data Aggregation Protocol for Wireless Sensor Networks

**Figure 3:**
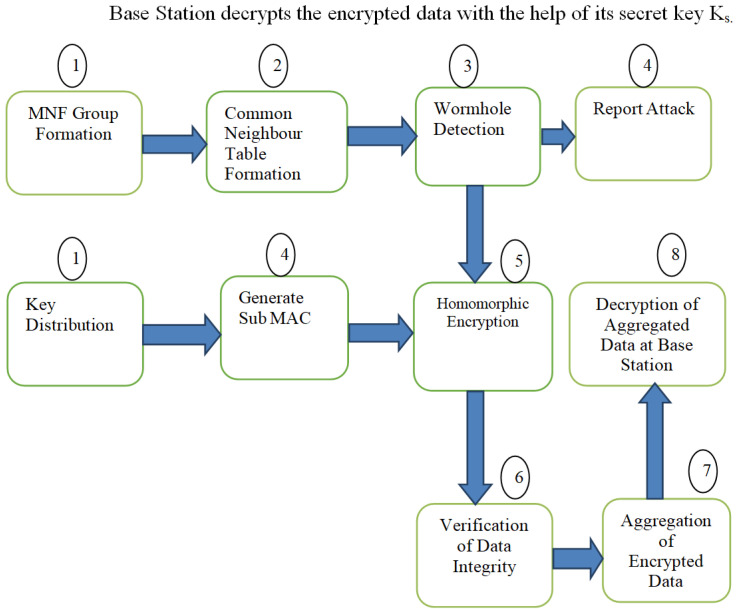
Flow Chart of the Proposed Algorithm.

**Figure 4:**
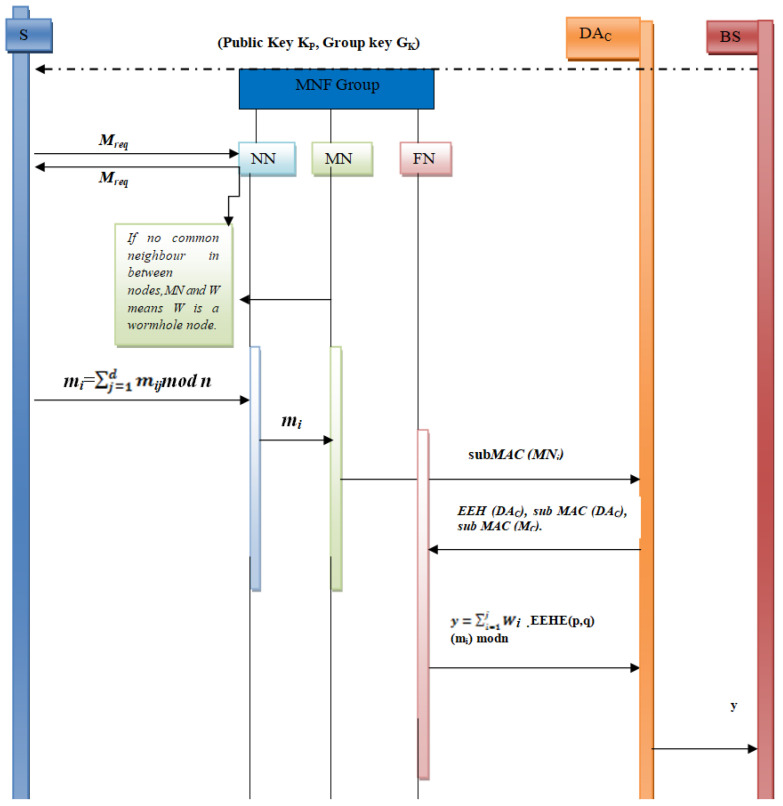
Timing diagram of the proposed work.

The proposed protocol (as illustrated in Proposed Algorithm 1) is based on the concept of a group of three sensor nodes designated as the monitoring node, neighbouring node and forwarding node. The group is called the MNF group. The flow of the proposed work can be understood from Figure 3 and the timing diagram shown in Figure 4. Detailed steps are as follows.

1. MNF Group Formation and Key Distribution. First of all, an MNF group consisting of three nodes (monitoring node, neighbouring node and forwarding node) is formed. The base station distributes a Gk to the MNF group and its public key to each node at the time of deployment of the sensor networks.
2. Common Neighbour Table (CNT) Formation. Information about common neighbours between the sender node, i.e., MN node and neighbour nodes is recorded in a table with the help of a CNT algorithm. This table will be helpful in detecting wormhole nodes.
3. Wormhole Detection. A wormhole node is detected with the help of common neighbour information between a sender and the neighbour node. There is a separate algorithm for wormhole detection, which will be explained in later sections.
4. Report Attack and Generate subMAC. An attack detection report is sent to the base station whenever a wormhole attack is detected. Now, the decision of isolation and removal is taken by the base station, which will be discussed in later sections. In order to verify the integrity of the message, a subMAC is generated by the monitoring node. This message subMAC (MNi) is sent to the DAc. Now, the end-to-end homomorphic value and subMAC EEH (DAc), subMAC (DAc), subMAC (MN) of the message are sent to the Forwarding Node (FN).
5. Homomorphic Encryption. Sender calculates the homomorphic value Mi = Mijmod n and sends it to the Neighbouring Node (NN). The Monitoring Node also receives this value and calculates a subMAC (MNi). This subMAC is then sent to the current DAc.
6. Verification of Data Integrity. DAc verifies the integrity of the data by recalculating the subMAC and sends the end-to-end homomorphic value and subMAC [node EEH(DAc), subMAC(DAc)] to a forwarding node (FN).
7. Aggregation of Encrypted Data. Now, the current DA computes the aggregated value i .EEHE(p,q)(mi) mod n and sends this value to the base station.
8. Decryption of Aggregated Data at Base Station. The Base Station decrypts the encrypted data with the help of its secret key Ks.

**Algorithm 1**: Proposed Algorithm.Input: - Readings of sensor nodesOutput: - Secure aggregated data transmissionStep 1: - MNF group consisting of three nodes (Monitoring node, Neighbouring Node and Forwarding node) is formed. Key distribution is also performed by Base Station.Step 2: - Common neighbour table of a MNF group is created by calling the CNT algorithm with request message Mreq and reply message Mrep.Step 3: - Check selected node is secure or not for transmission. Call Algorithm Wormhole Detection.Step 4: - If wormhole is detected, an error is reported to the Base Station;
else, go to step 5.
- SubMAC is generated by the monitoring node for data integrity check.Step 5: - EEHE is performed by the sender node with the help of the public key of Base Station to ensure confidentiality of data.Step 6: - Data integrity is verified by the neighbouring node by recalculation of MAC.Step 7: - Aggregation of encrypted data is performed by DA node.Step 8: - Base station decrypts the aggregated and encrypted data with the help of its secret key.

### 4.1. Message Authentication Code (MAC)

Let Dest, AM, Len, and PNum represent the destination address, active message type, message length, and packet sequence number, respectively. A MAC is used by monitoring nodes to validate data integrity. A symmetric key is shared by the two subsequent aggregators. Every MAC packet of data comprises the source address (2 bytes), destination address (2 bytes), AM (1 byte), LEN (1 byte), PNum (1 byte), and end-to-end homomorphic data (0–29 bytes). The MAC message format is a kind of logical cross-layer packet structure. Table 2 shows the packet structure of our design. Whenever groups are established but each grouping node does have the same pseudorandom sequence generator, data synchronization is not accomplished because of cipher text losses. A sequence number is appended to each aggregated data packet to ensure synchronization. The order of the subMACs is established by the aggregator, and each monitoring node is informed of its own subMAC position.

#### Common Neighbour Table

The CNT (Common Neighbour Table) algorithm describes the formation of a common neighbour table between a monitoring node (MN) and a neighbouring node (NN). In the first step, node MN, as the sender node, broadcasts a request message Mreq to its neighbour nodes NN, which are present in its communication range. Each neighbour node NN receives the message and sends a reply message Mrep back to the monitoring node MN. Once an MN node receives a response message, it modifies its neighbour database. We will use this neighbour table to find a common neighbour between the MN and NNs.

### 4.2. Data Aggregation and Integrity Detection

To maintain the EEHE feature, when such data are transmitted to the aggregator, the DA immediately aggregates the data without decoding it. The MNF group is made up of a KP and a tuple of cipher text (CT1, CT2, CT3……………CTN). With those input values, a separate cipher text outcome is generated. The output value is recorded in the variable O.

O = encrypt (KP, C, CT1, CT2, CT3……CTN).

Decryption (KS, O) = C (M1, M2, M3, M4……MN).

To ensure data integrity, privacy, and wormhole identification throughout the data aggregation and forwarding process, a data integrity and data aggregation method is designed. In the process, the DA of one MNF group confirms the subMAC generated by the DA of the other MNF group. If the subMAC verification succeeds, the data are marked; alternatively, the packet of data is rejected. After the data are marked, step 1 of the algorithm is performed to ensure the integrity and aggregation of the data. This procedure is continued until all the MNF group nodes and DAs have been addressed. In this section, wormhole detection using the wormhole detection Algorithm 2 under a given scenario is discussed. If there is a false node in the network, then its information is passed to the source nodes.
**Algorithm 2:** Wormhole Detection (WD) Algorithm.Input: - MN, NN, CNT.Output: - Secure Data Aggregation.Step 1: - MN broadcasts Mreq to NN.Step 2: - NN receives Mreq and sends Mrep to MN.Step 3: - If there is a wormhole node W, then it sends Mrep with fake node ID and fake location.Step 4: - There will be two cases:Step 5: - In case 1, if W does not have the neighbours’ ID, MN will confirm CNT in between MN and W nodes.Step 6: - If there are no common nodes, it means W is a wormhole node.Step 7: - In case 2, if W has the neighbours’ ID, MN will confirm the CNT in between nodes MN and W.Step 8: - Common nodes between MN and node W confirm CNT.Step 9: - If any node has encountered the ID of a suspicious W in its table, then node W is declared as the trusted one.Step 10: - Else, transmission is stopped.

## 5. Security Analysis and Experimental Results

In the proposed method, the aggregated data meet the security criteria of secrecy, data integrity, wormhole attack detection, and false data identification.

### 5.1. Data Confidentiality

Data confidentiality in WSNs implies that only the intended receiver and the sender will be able to know the data and they will never be disclosed to an unauthorized node. As a result, our approach is resistant to cipher-text-only threats. Even though the aggregated data are revealed, the opponent could only access the aggregation output, not the sensor readings. Furthermore, it is robust to plain-text-only threats. In 1994, the American mathematician Peter Shor devised a polynomial-time quantum method for factorization [19]. Until quantum computers become available, generic range fields sieving has been the quickest recognized conventional approach to addressing an implementation of the factorization challenge. Therefore, for the future, it is safe to rely on the RSA’s encryption.

### 5.2. Data Integrity

Data integrity ensures that none of the data are manipulated throughout the network communication process. While confidentiality ensures that only the designated parties receive non-encrypted plain data, it does not protect data from being manipulated. In the proposed scheme, 6 bytes are used for MAC in a data packet. The security of a 6-byte MAC can be broken in 26 × 8 trials, whereby an attacker does have a 1 in 26 × 8 possibility of counterfeiting the MAC code. Although extending the length of the MAC adds to the computational cost, such a scheme employs a subMAC with a size of 32/(N + 1) bits, in which N represents the amount of nodes in a specific cluster, and N + 1 subMACs calculated by N + 1 nodes constitute a MAC. As a result, an adversary may effectively duplicate a genuine MAC if it identifies every N + 1 subMACs with a 1 in 248/(N + 1) chance for every subMAC. Therefore, the chance that the MAC will not identify the fake data is (1/248/(N + 1))N + 1 = 1/248.

### 5.3. Wormhole Attack Detection and False Positives

The presented protocol’s performance is comparable to method [4], the key concept of which is that each sensor node calculates the connectiveness level of its peers and reports the existence of the wormhole while using parameters. This section discusses the key phases of this procedure.

Supposition: In a network composed like a WSN, it is considered that each sensor node has a minimum of one shared 1–2 hop neighbours.

1. Neighbour discovery: Each node keeps track of its 1 or 2 hop neighbours.
2. Computing: Every node initially evaluates their clustering coefficient.
3. Isolation: When a node is labeled a wormhole, the voting procedure is implemented. A generalization of the scheme is: if X is l-hop away from node a, a declares X as a wormhole if [3]

(1) $$\exists k\in V_{1}\left(X\right)s.t.C_{a,k/X}^{L+2}=0$$

**Figure 5:**
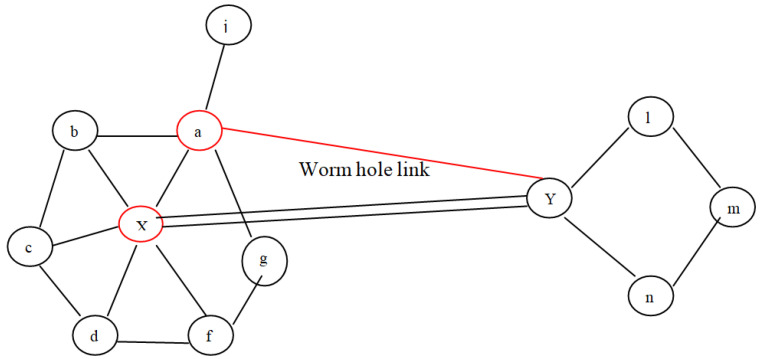
Wormhole attack detection example.

**Figure 6:**
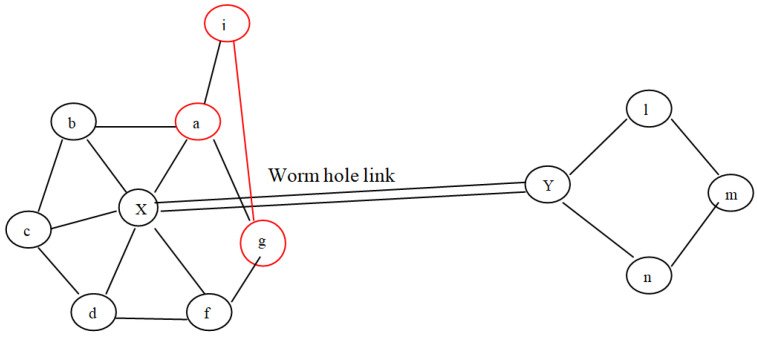
Example of existence of false positive.

As aforementioned, wormhole deployment entails establishing a long connection across two wormhole nodes. Almost all the time, the connection is wide enough to provide the lowest pathways across two groups of potentially distant nodes. Therefore, as from the perspective of graphs, a wormhole connection yields misleading neighbouring knowledge among nodes which assume that there are k-hop peers but actually there are not. To be more precise, the wormhole link assumes that two wormhole nodes, say X and Y, are I-hop neighbours even when they are not. Furthermore, if both nodes were identified as having I-hop neighbour, as well as the network seeming highly dense, then these two particular nodes have some shared I-hop neighbour, which is not the scenario if the associated route is really a wormhole. Consider the situation represented in Figure 5, wherein node a considers node X to be a wormhole because it did not fulfil the constraints mentioned above under Equation (1). In the second scenario, as illustrated in Figure 6, the requirement is fulfilled; however, there are false positives. In the proposed protocol, the common neighbour table is created in order to detect a wormhole. Due to the overhearing property of wireless communication, common neighbours between a sender and a receiver decide whether the receiver is a wormhole or not. Thus, there is no chance of the occurrence of any false positive. In the proposed technique, the last step of the given protocol, i.e., voting, can be avoided. The proposed scheme requires less energy as compared to the protocol discussed, and there are lower chances of false positives, which will be demonstrated by experimental results in the next section.

### 5.4. Identification and Removal of All Wormhole Nodes in the Network

Two types of nodes can be defined neighbouring the wormhole area. One may be affected nodes and another may be unaffected nodes. Unharmed nodes are those which are beyond the transmission radius/range of the wormhole nodes. Vulnerable nodes are those whose neighbour tables are altered as a result of the existence of the wormhole link. The approach identifies a group of malicious/malignant nodes, which comprises both affected nodes as well as some unaffected nodes. On the other side, all nodes that are not detected by this approach are unaffected nodes that do not contain wormhole-created spurious connections. To eradicate the wormhole connections, every destructive node W finds the intersection of its neighbourhood table NT (W) from the neighbourhood tables of its non-affected peers/neighbours. Node W blacklists any node x in NT (W) that is not part of any such intersections. Node x blocks any future broadcasts from these kinds of nodes, proving the wormhole attack unsuccessful. Once all suspected nodes have concluded blacklisting nodes from their neighbour listings, wormhole elimination is achieved.

### 5.5. Performance Evaluation of Proposed Algorithm

In this section, an evaluation of the proposed algorithm that screens a WSN for a wormhole attack is performed on the basis of parameters such as sensitivity, specificity, likelihood ratio, predictive value etc. as illustrated in Table 3. This algorithm detects whether the given node is a wormhole or not.

1. Sensitivity. The outcome of the algorithm can be positive (predicting that the node is a wormhole) or negative (predicting that the node is not a wormhole). Mathematically, sensitivity [20,21] can be expressed as in Equation (2).
  (2) $$Sensitivity=\frac{\alpha }{\alpha +\gamma },$$
  where α is the count of true positives and γ is the count of false negatives.
2. Specificity. Mathematically, specificity can also be written as shown in Equation (3) [20,21].
  (3) $$Specificity=\frac{\delta }{\beta +\delta },$$
  where β is the number of false positives, and δ is the number of true negatives.
3. Positive likelihood ratio: as shown in Equation (4).
  (4) $$Positive\;likelihood\;ratio=\frac{True\;positive\;rate}{False\;positive\;rate}=\frac{Sensitivity}{1-Specificity}$$
4. Negative likelihood ratio: as shown in Equation (5).
  (5) $$Positive\;likelihood\;ratio=\frac{False\;positive\;rate}{True\;positive\;rate}=\frac{1-Sensitivity}{Specificity}$$
5. Positive predictive value: as shown in Equation (6).
  (6) $$Positive\;predictive\;value=\frac{\alpha }{\alpha +\beta }$$
6. Negative predictive value: as shown in Equation (7).
  (7) $$Negative\;predictive\;value=\frac{\beta }{\beta +\delta }$$

## 6. Experimental Results

To measure the recommended method’s performance, it is simulated utilizing OMNET++, and its performance across varying conditions is analyzed. Quantity matrices comprising mean power dissipation, network lifespan, and the proportion of surviving nodes are used to evaluate performance. Across the simulation, a network topology of 100 nodes is considered, with every node possessing an initial energy of 2 Joules. In the first simulation, a comparison of the proposed protocol with an existing protocol [3,4], in terms of the probability of false positives and probability of wormhole detection, is performed. In the second simulation, a comparison of the proposed protocol’s average energy dissipation and system lifetime with the existing protocol is performed.

### 6.1. Probability of False Positives and Wormhole Detection

**Figure 7:**
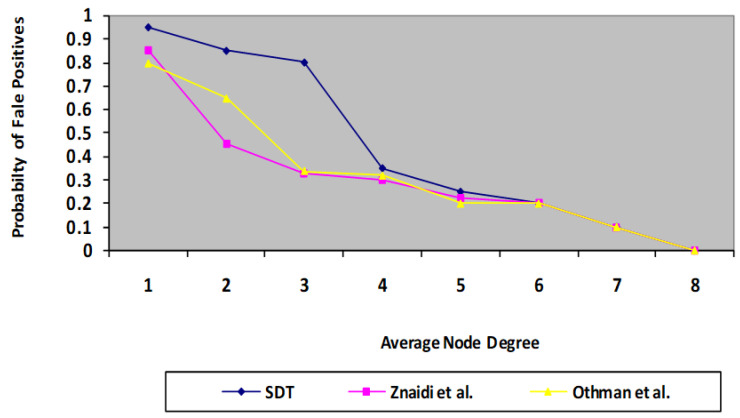
Comparison of proposed SDT protocol with existing protocol in terms of probability of false positives.

**Figure 8:**
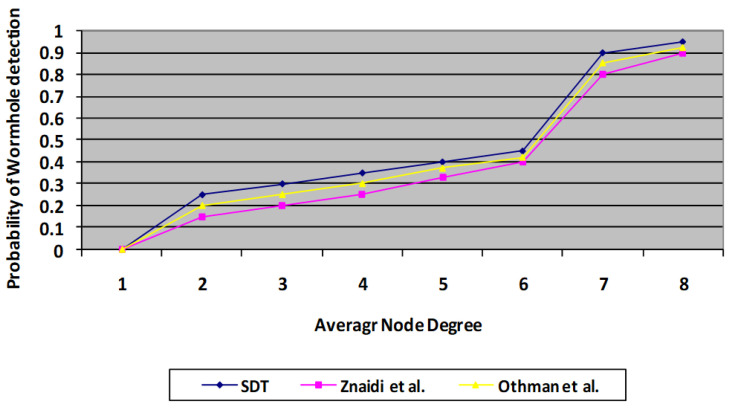
Comparison of proposed SDT protocol with existing protocol in terms of probability of wormhole detection.

The algorithm is run through its tests employing a randomized distribution. Every simulation is conducted using approximately 100 sensor nodes dispersed over a 400 * 400 m square field with just one wormhole. During every experiment, the deployed nodes maintain fixed positions. The speed protocol is applied for route tasks. Thus, node degree is taken as an important parameter for evaluation. Whenever node degree increases, the probability of false positives decreases. Whenever node degree increases, it means the number of MNF groups in a network also increases. Consequently, the monitoring mechanism by neighbours increases. Thus, the chances of false positives decrease compared with the existing scheme. A similar situation occurs in the case of the probability of wormhole detection. The findings indicate the mean of 150 cycles, having the identical connection model with randomized constructed topologies. In each test, a single wormhole threat is mounted at random, involving two nodes separated by more than four hops. With this technique, two probabilities are estimated for every set of rounds: the risk of a false positive and the chance of detection, as illustrated in Figure 7 and Figure 8, where the results of the presented protocol are proven to be better than those of the previous protocols [3,4].

### 6.2. Average Energy Dissipation

**Figure 9:**
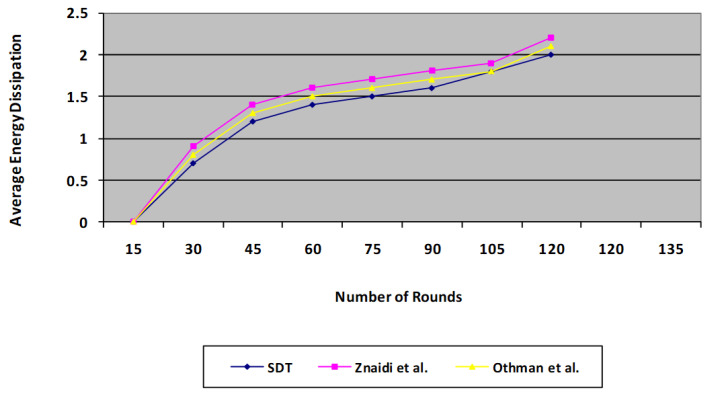
Comparison of proposed SDT protocol’s Average Energy Dissipation with the existing protocol.

Figure 9 depicts the protocol’s mean power dissipation across the number of iterations of operations. The graph clearly reveals that the proposed SDT protocol has a significantly superior energy expenditure slope to that of the existing protocol [3,4]. On average, the proposed technique consumes 30% less power than the existing approach. This happens due to the fact that the data aggregation process reduces the number of message transfers. When wormhole attack detection is used, there exists a small rise in power consumption, as transmission takes more energy than computation in the sensors. Our proposed protocol is found to be more energy efficient compared with the existing protocol, as it omits a voting phase which consumes a substantial amount of energy due to a higher number of message transfers.

### 6.3. System Lifetime

**Figure 10:**
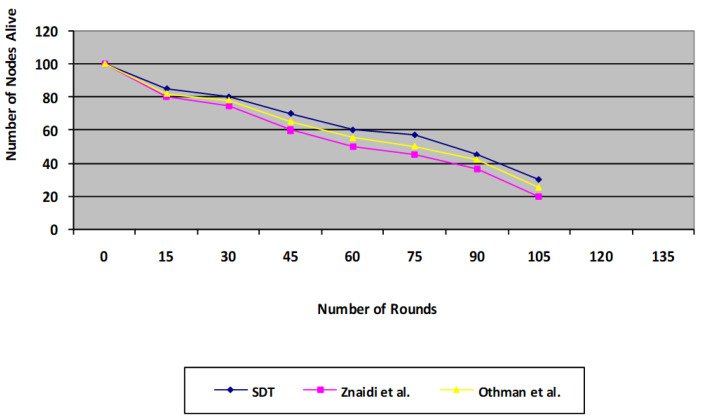
Comparison of proposed SDT protocol’s System Lifetime with the existing protocol.

The system lifespan curve in Figure 10 additionally highlights the improvements achieved with the proposed protocol. For the 100 * 100 m network scenario model, this plot depicts the amount of nodes that remain live across a number of iterations of activity. For the recommended SDT protocol, 60% of the nodes continue living over 60 rounds, whereas 50% of the nodes remain alive for the existing protocol. Therefore, in the proposed method, 20% of the nodes remain live for 120 rounds; however, the correlation for the existing protocol was 0 live nodes, in other words, all nodes were dead for the existing protocol without aggregation at 105 rounds. Our proposed protocol is found to be more energy efficient compared with the existing protocol, as it omits the voting phase in the existing technique, which consumes a substantial amount of energy due to the number of message transfers.

### 6.4. Aggregation Accuracy

**Figure 11:**
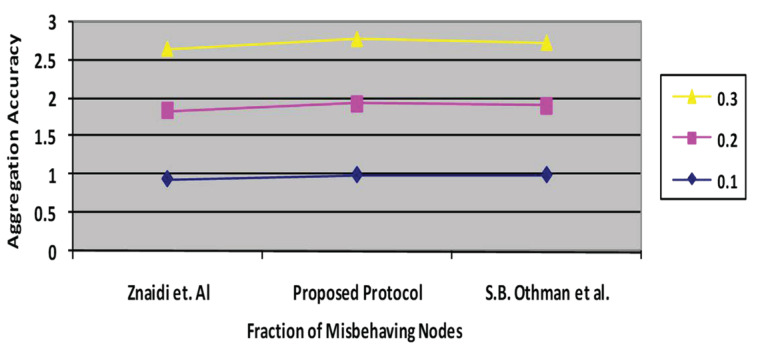
Aggregation accuracy vs. percentage of fractions of misbehaving nodes.

Aggregation accuracy rapidly declines as the number of malicious nodes grows. Figure 11 depicts the aggregation accuracy of the proposed approach and the existing protocol [3,4]. Typically, the accuracy diminishes as the proportion of malfunctioning nodes increases. The percentage of malfunctioning nodes varied from 0% to 40%. Whenever it rises, refs. [3,4] indicates 0.67, and the proposed protocol exhibits 0.69.

## 7. Conclusions

In this paper, a technique is described that ensures confidentiality in data fusion between a transmitter and receiver and also identifies wormhole threats on a specific node utilizing the shared neighbours table. In the proposed protocol, a common neighbour table is created in order to perform an integrity check and detect a wormhole. Due to the overhearing property of wireless communication, common neighbours between a sender and a receiver decide whether the receiver is a wormhole or not. Thus, there are fewer chances of false positive results. The identification and removal of all wormhole nodes in the network are also discussed in the proposed protocol. An EEHE technique is used for achieving end-to-end confidentiality of the aggregated data. The proposed scheme requires less energy compared with the protocol [3,4], and there are fewer chances of false positives. The key features of the SDT technique are that it does not require any guard nodes or special hardware, and there is no data loss owing to wormhole events. Whenever a false node is discovered, an alert alarm is triggered without any additional directional antennas. In the present work, neighbouring information, particularly node ID, is used to detect the presence of wormholes. This scheme fails whenever there exists a malicious node with a fake node ID which acts as a trustworthy node and misleads the routing protocol. In future, the location-based wormhole attack detection protocol could be taken into account.

## Figures and Tables

**Table 1:** Comparisons of various End-to-End Secure Data Aggregation Protocols.

Protocol	Data Confidentiality	Data Integrity	Source Authentication	Node Availability	Prevention of Attacks	E-E Security/ H-H Security	Aggregation Function	Techniques Used
Hung et al. [16], 2008	Yes	Yes	No	No	Snooping, identification of malicious nodes	E-E	SUM	Homomorphic Encryption, Digital Signature
SEEDA et al. [10], 2009	Yes	No	No	No	Eavesdropping	Both	SUM AVERAGE	Homomorphic Encryption
Jacques et al. [17], 2010	Yes	No	No	No	Man in the middle attack, recognized simple text threat, and targeted plain text invasion	E-E	SUM	Homomorphic encryption, elliptic curve cryptography
IPHCDA [12], 2011	Yes	Yes	No	No	Snooping, replay attacks, recognized plain text attacks, encrypted analytics, illegal aggregation	E-E	SUM	Homomorphic encryption, MAC
Suat Ozdemir, and Hasan Çam [11], 2010	Yes	Yes	Yes	No	Sybil, replaying, fake information discovery, snooping	E-E	SUM	MAC, Group Key Management
EEHA [13], 2011	Yes	Yes	No	No	Eavesdropping, replaying attack	E-E	SUM	MAC
SEDA-ECC [14], 2014	Yes	No	No	No	Node Compromised	E-E	SUM	FHE, MAC
FESA [15], 2015	Yes	Yes	Yes		False data injection	E-E	SUM	ECC and Divide and Conquer
S. B. Othman et al. [4]	Yes	Yes	No	No	False data injection	E-E	SUM	Homomorphic encryption based on symmetric key cryptography, MAC

**Table 2:** MAC Packet Strucure.

Dest (2)	AM (2)	Len (1)	Data (0–29)	PNum (1)	MAC (6)

**Table 3:** Comparison of the proposed SDT with the existing protocols in terms of sensitivity and specificity of wormhole attack detection.

Parameters	Proposed Algorithm	Znaidi et al. [3]	Othman et al. [4]
Sensitivity	66.67%	50.00%	63.69%
Specificity	90.91%	85.71%	89.77%
Positive Likelihood Ratio	7.33	3.50	4.31
Negative Likelihood Ratio	0.37	0.58	0.40
Attack prevalence	21.43%	22.22%	20.18%
Positive Predictive Value	66.67%	50.00%	58.69%
Negative Predictive Value	90.91%	85.71%	88.77%

## Data Availability

No data are associated with this study.
